# Insights Into Forensic Features and Genetic Structures of Guangdong Maoming Han Based on 27 Y-STRs

**DOI:** 10.3389/fgene.2021.690504

**Published:** 2021-06-18

**Authors:** Haoliang Fan, Qiqian Xie, Yanning Li, Lingxiang Wang, Shao-Qing Wen, Pingming Qiu

**Affiliations:** ^1^School of Forensic Medicine, Southern Medical University, Guangzhou, China; ^2^Institute of Archaeological Science, Fudan University, Shanghai, China; ^3^School of Basic Medicine and Life Science, Hainan Medical University, Haikou, China; ^4^School of Basic Medicine, Gannan Medical University, Ganzhou, China

**Keywords:** Maoming Han, Gaoliang culture, Y-STR, forensic features, genetic structures

## Abstract

Maoming is located in the southwest region of Guangdong Province and is the cradle of Gaoliang culture, which is the representative branch of Lingnan cultures. Historical records showed that the amalgamations between Gaoliang aborigines and distinct ethnic minorities had some influences on the shaping of Gaoliang culture, especially for the local Tai-kadai language-speaking Baiyue and Han Chinese from Central China. However, there is still no exact genetic evidence for the influences on the genetic pool of Maoming Han, and the genetic relationships between Maoming Han and other Chinese populations are still unclear. Hence, in order to get a better understanding of the paternal genetic structures and characterize the forensic features of 27 Y-chromosomal short tandem repeats (Y-STRs) in Han Chinese from Guangdong Maoming, we firstly applied the AmpFLSTR^®^ Yfiler^®^ Plus PCR Amplification Kit (Thermo Fisher Scientific, Waltham, MA, United States) to genotype the haplotypes in 431 Han males residing in Maoming. A total of 263 different alleles were determined across all 27 Y-STRs with the corresponding allelic frequencies from 0.0004 to 0.7401, and the range of genetic diversity (GD) was 0.4027 (DYS391) to 0.9596 (DYS385a/b). In the first batch of 27 Yfiler data in Maoming Han, 417 distinct haplotypes were discovered, and nine off-ladder alleles were identified at six Y-STRs; in addition, no copy number variant or null allele was detected. The overall haplotype diversity (HD) and discrimination capacity (DC) of 27 Yfiler were 0.9997 and 0.9675, respectively, which demonstrated that the 6-dye and 27-plex system has sufficient system effectiveness for forensic applications in Maoming Han. What is more, the phylogenetic analyses indicated that Maoming Han, which is a Southern Han Chinese population, has a close relationship with Meizhou Kejia, which uncovered that the role of the gene flows from surrounding Han populations in shaping the genetic pool of Maoming Han cannot be ignored. From the perspectives of genetics, linguistics, and geographies, the genetic structures of Han populations correspond to the patterns of the geographical-scale spatial distributions and the relationships of language families. Nevertheless, no exact genetic evidence supports the intimate relationships between Maoming Han and Tai-Kadai language-speaking populations and Han populations of Central Plains in the present study.

## Introduction

**FIGURE 1:**
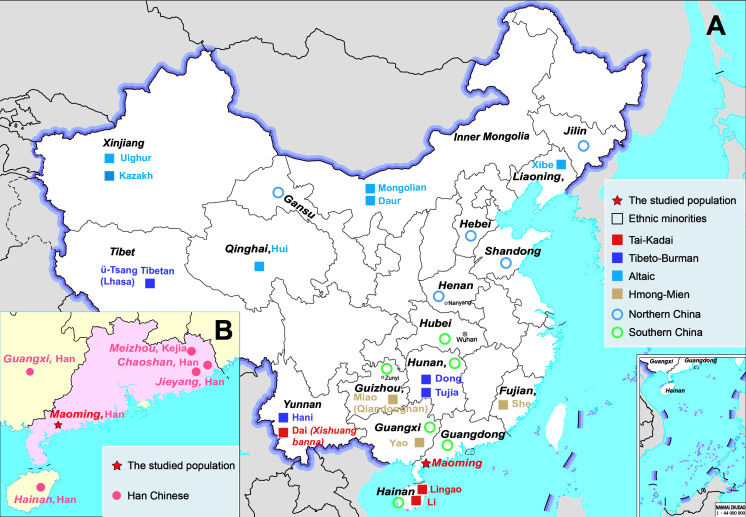
Geographical locations of population distributions and sampling information. **(A)** The geographical distribution of Maoming Han and other Chinese populations (Han Chinese and ethnic minorities), which was analyzed in the present study. **(B)** The geographical distribution of Maoming Han and other five Han Chinese populations in South China (our studied group is marked as a red pentagram).

Maoming, a city located in the southwest of Guangdong Province (Figure 1), is the cradle of Gaoliang culture (Zhou, 2019). Gaoliang culture, one of the representative Lingnan cultures, could be dated back to the Han Dynasty (111 B.C.) in Chinese history (He, 2012). The aborigines living in Gaoliang mountainous areas and the basins between Jian River and Moyang River are the inheritors of Gaoliang culture, which are represented by the customs of *Nianli* (a special celebration for New Year) and *Piaose* (a form of dramatic plastic arts on moving stages) (Chen, 2013). Since the Southern and Northern Dynasties (420–589 A.D.), the intermarriages accelerated national amalgamations between Gaoliang aborigines and other ethnic minorities in ancient Gaoliang District (He, 2012; Chen, 2013)12). Therefore, Gaoliang culture was influenced by the convergences between Gaoliang aborigines and different ethnic groups (Gao, 2007). Moreover, some archeological records also hinted that the population structures of Gaoliang aborigines might be affected by the local Baiyue (a Tai-kadai language-speaking population in ancient China) and Han Chinese from Central China with the increasingly social activities of mixed marriages, population migrations, and trade contacts in the long course of history (Gao, 2007). Maoming Hans, the descendants of Gaoliang aborigines, speak Cantonese (*Gaoyang Pian*), which is one branch of Sino-Tibetan language family (Ding, 2010). From the perspective of languages, the language of Maoming Han (Cantonese) did not seem to be impacted by Tai-Kadai groups (Baiyue). Hence, there is still no exact genetic evidence for the influences on the genetic pool of Maoming Han, and the genetic relationships between Maoming Han and other surrounding populations are still unclear.

Y-chromosomal short tandem repeats (Y-STRs) have been regarded as a valuable tool in forensic genetics (Kayser, 2017), genealogy (Kayser et al., 2007), human evolution (Underhill and Kivisild, 2007), archeology (Calafell and Larmuseau, 2017), population history (Kayser et al., 2000; Jobling and Tyler-Smith, 2017), and male medical genetics (Hughes and Page, 2016; Jobling and Tyler-Smith, 2017). In addition, Y chromosomal variant analysis for determining the patterns of present and past flows of genes between populations is very helpful (Oppenheimer, 2012). The use of Y-STRs also allows the simultaneous analysis of closely related and distantly related populations (Ballantyne and Kayser, 2012). The 6-dye and 27-plex AmpFLSTR^®^ Yfiler^®^ Plus PCR Amplification Kit (Thermo Fisher Scientific, Waltham, MA, United States) includes 17 Yfiler loci (DYS19, DYS385a/b, DYS389I/II, DYS390, DYS391, DYS392, DYS393, DYS437, DYS438, DYS439, DYS448, DYS456, DYS458, DYS635, and Y GATA H4) plus three highly polymorphic Y-STR loci (DYS460, DYS481, and DYS533) and seven rapidly mutating Y-STR loci (DYF387S1a/b, DYS449, DYS518, DYS570, DYS576, and DYS627) in an effort to improve discrimination of related individuals (Gopinath et al., 2016). Y-chromosome STR haplotype reference database (YHRD)^1^ is an internet-accessible worldwide reference database of Y chromosome profiles, which contributed to provide a worldwide and high-quality Y-STR haplotype data from distinct human populations for forensic purposes and population genetics (Willuweit and Roewer, 2015). As yet, little is known about the genetic backgrounds of the aforementioned 27 Y-STRs in Maoming Han, and the forensic-related Y chromosome variation data in Guangdong Maoming still remains blank in YHRD.

Hence, in order to get a better understanding of the paternal genetic structure and characterize the forensic resolution of 27 Y-STRs in Han Chinese from Guangdong Maoming, we used the 6-dye and 27-plex Y-STR system to genotype the haplotypes in 431 Han males residing in Maoming city. Furthermore, we explored the genetic relationships between Maoming Han and Chinese populations of Southern and Northern China from the perspectives of geographies, linguistics, and genetics.

## Materials and Methods

### Sample Preparation

In this study, a total of 431 unrelated Han Chinese males were recruited from Maoming city, Guangdong Province, China (Figure 1). The inclusion criteria were as follows: (1) healthy individuals without any underlying diseases (including but not limited to cardiovascular diseases, metabolic diseases, chronic wasting diseases, immunologic diseases, etc.); (2) unrelated males and any two individuals who have no blood relationship for up to three generations; (3) the volunteers’ parents and grandparents are aboriginals and have non-consanguineous marriages of the same ethnic group for at least three generations, which was confirmed by the volunteers’ self-declared statements; and (4) the language Cantonese is the mother tongue of Maoming volunteers, and any self-declared Maoming Han who could not speak Cantonese would be excluded from our cohort. Blood samples of all Maoming volunteers were collected using FTA cards (Whatman^TM^, GE Healthcare, Chicago, IL, United States) with written informed consents from participants. All the experimental procedures were performed following the standards of the Declaration of Helsinki. This study was approved by the Medical Ethics Committee of Hainan Medical University (no. HYLL-2020-012).

### DNA Extraction, Amplification, and Genotyping

Genomic DNA was extracted using the TIANamp Blood Spot DNA Kit (TIANGEN BIOTECH, Beijing, China) according to the manufacturer’s protocol. The quantity of the DNA templates was determined using Qubit^TM^ dsDNA HS Assay Kit (Thermo Fisher Scientific, Waltham, MA, United States) on the Qubit 4.0 Fluorometer (Thermo Fisher Scientific, Waltham, MA, United States) according to the manufacturer’s instructions. Based on the quantitative results, DNA samples were normalized to 2.0 ng/μl and stored at −20°C until amplification.

The amplification of the 6-dye multiplex PCR-CE-based AmpFLSTR^®^ Yfiler^®^ Plus PCR Amplification Kit (Thermo Fisher Scientific, Waltham, MA, United States) was performed in a single multiplex PCR reaction (25 μl in total, containing 10 μl master mix, 5 μl primer mix, and 10 μl genomic DNA) on a Veriti^®^ 96-Well Thermal Cycler System (Thermo Fisher Scientific, Waltham, MA, United States) following the manufacturer’s instructions. Amplified products were separated by capillary electrophoresis (CE) on a 3500xL Genetic Analyzer (Thermo Fisher Scientific, Waltham, MA, United States). The separation of CE-based amplified products was conducted according to our previous studies (Fan et al., 2019a; Liu et al., 2020).

### Statistic and Population Genetic Analyses

Allele and haplotype frequencies as well as forensic parameters were calculated using direct counting. The relevant forensic parameters contained genetic diversity (GD), haplotype diversity (HD), discrimination capacity (DC), and random match probability (RMP). GD was calculated according to the following formula:

GD=nn-1×(1-pi2)

where *n* is the total sample size, and *p_i* indicates the frequency of *i*-th allele. HD was computed in the same formula as GD, except that *p_i* refers to the frequency of *i*-th haplotype. DC is equal to the ratio of different haplotypes to the total sample size. Computed with the formula RMP=∑pi2, RMP is the probability that a particular DNA profile would appear in a population and that a “match” would occur by coincidence. In forensic statistics, a lower RMP value indicates higher strength of evidence provided by genetic analysis.

Population pairwise genetic distance (*R*_*st*_) is commonly used for estimating the population differences and computing the genetic relationships among different populations (Fan et al., 2019b; Li et al., 2020). By using the “AMOVA&MDS tool” on YHRD, pairwise *R*_*st*_ and corresponding *p* values based on 17 Yfiler between Maoming Han and reference populations were estimated by analysis of molecular variance (AMOVA) and visualized in multidimensional scaling (MDS) plot, which were used show the reduced dimensionality spatial representation of the populations. Additionally, phylogenetic relationships among Han Chinese populations from Southern and Northern mainland China as well as those between 6 Han Chinese and 16 ethnic minorities were depicted in the Molecular Evolutionary Genetics Analysis-X (MEGA-X) software (Kumar et al., 2018) by a neighbor-joining (N-J) phylogenetic tree (Saitou and Nei, 1987) based upon the *R*_*st*_ genetic distance matrix, respectively.

### Quality Control

The recommendations of the DNA Commission of the Chinese National Standards, the Scientific Working Group on DNA Analysis Methods (SWGDAM) (SWGDAM, 2010), and the DNA Commission of the International Society of Forensic Genetics (ISFG) (Gusmao et al., 2006; Carracedo et al., 2013; Roewer et al., 2020) for analysis of Y-STRs were strictly followed. Control DNA 007 was employed as a positive control, while ddH_2_O was used as a negative control for each batch of amplification and genotyping. Additionally, the laboratory has passed the proficiency testing for Y-STR typing organized by YHRD and has been accredited in accordance with ISO/IEC 17025:2005 and the China National Accreditation Service for Conformity Assessment (CNAS). The haplotype data of 431 unrelated male individuals from Guangdong Maoming Han population in the present study have been submitted to YHRD database and received the accession number YA004720 (Maoming Han, *n* = 431). The Y-STR profiles with off-ladders were re-amplified and re-genotyped by Goldeneye DNA^TM^ ID 27YB system (Goldeneye^®^ Technology Ltd., Beijing, China).

## Results and Discussion

In the present study, a total of 431 unrelated male individuals from Han Chinese in Guangdong Maoming were genotyped including 27 Y-STR loci using the AmpFLSTR^®^ Yfiler^®^ Plus PCR Amplification Kit (Thermo Fisher Scientific, Waltham, MA, United States). In order to evaluate the forensic features of Maoming Han population, we set up two datasets, Yfiler set and Yfiler Plus set, including 17 and 27 Y-STRs, respectively. In addition, a series of comprehensive population genetic analyses were conducted between Maoming Han and other southern and northern Chinese populations. In short, the aims of this study were to feature the forensic characteristics of 27 Y-STRs in Maoming Han, clarify the paternal genetic structures of Maoming Han, and get a better understanding of the genetic relationships between Maoming Han and other Chinese populations from the perspectives of geographics, linguistics, and genetics.

### Forensic Characteristics

#### Forensic Features of Yfiler Set (17 Y-STRs)

As illustrated in Supplementary Table 1, a total of 147 distinct alleles were identified across all 17 Y-STRs in Maoming Han with the corresponding allelic frequencies from 0.0023 to 0.7401 (DYS391). Overall, 17 Yfiler loci were relatively highly polymorphic in Maoming Han. The range of allele numbers was 4 (DYS391, DYS437, and DYS438) ∼ 55 (DYS385a/b), and the lowest and highest estimates of GD corresponded to loci DYS391 (0.4027) and DYS385a/b (0.9596). Except for DYS391 (0.4027) and DYS438 (0.4049), the GD values for other 17 Yfiler loci were greater than 0.5. The haplotypes and haplotype frequencies of Yfiler in Maoming Han are shown in Supplementary Table 2. There were 371 different haplotypes observed in 431 Maoming Han individuals, of which 328 (88.41%) were unique, 33 occurred twice (S011–S043), 6 (S005–S010) were observed thrice, 1 (H004) was shared by 4 individuals, and 3 (S001–S003) were shared by 5 individuals. We observed four confirmed microvariants [18.2 (twice) and 19.2 at DYS448 and 18.2 at DYS458]. The overall HD was 0.9994 with a DC of 0.8608.

#### Forensic Features of Yfiler Plus Set (27 Y-STRs)

Allele frequency distributions and haplotype frequencies of Yfiler Plus for Maoming Han are presented in Supplementary Tables 3, 4. A total of 263 different alleles were observed, and the number of distinct alleles ranged from 4 for DYS391, DYS437, and DYS438 to 55 for DYS385a/b. Allele frequencies varied from 0.0004 to 0.7401. All 10 newly added loci got GD values higher than 0.5, especially for the added multi-copy DYF387S1a/b (0.9682). DYS385a/b (0.9596) on the one hand while DYS391 (0.4027) and DYS438 (0.4049) on the other marked the extremes of the GD distribution (with GD values less than 0.5). Genotyping with the 27 Y-STRs determined 417 distinct haplotypes in the population of Maoming Han, of which 405 (97.12%) were unique, 10 different haplotypes were identified twice (H003–H012), and 2 (H001-H002) appeared thrice. In addition to 18.2 and 19.2 at DYS448 and 18.2 at DYS458, intermediate alleles were also observed at the DYS449 (34.2), DYS518 (37.2), DYF387S1 (37.2), and DYS627 (17.2 and 18.2) loci. The overall HD and DC were calculated to be 0.9997 and 0.9675, respectively.

In this study, duplicated or triplicated alleles and null alleles were not detected in both Yfiler set and Yfiler Plus set. The analysis of genotype data revealed that DYS385a/b and DYF387S1a/b showed higher GD in Maoming Han, which were the same as other Chinese populations (Fan et al., 2018a). Forensic parameters based on different sets of Y-STR loci were calculated and listed in Table 1, indicating that as the number of Y-STR loci increased, more distinct haplotypes were identified, and HD and DC were also increased in the present study.

**TABLE 1:** Forensic features of Yfiler and Yfiler Plus loci in 431 Maoming Han.

**Number of observed haplotypes**	**Y-filer (17Y-STR)**	**Y-filer Plus (27Y-STR)**
1 (unique)	328	405
2	33	10
3	6	2
4	1	–
5	3	–
Sample size	431	431
Number of unique haplotypes	371	417
Proportion of unique haplotypes	0.8841	0.9712
HD	0.9994	0.9997
DC	0.8608	0.9675
RMP	0.0033	0.0027

*HD, haplotype diversity; DC, discrimination capacity; RMP, random match probability.*

### Genetic Differences Between Maoming Han and Han Chinese Populations From Southern and Northern Mainland China

**FIGURE 2:**
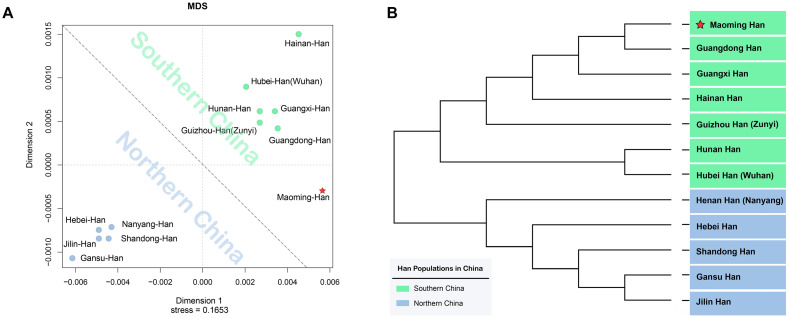
Genetic similarities and differences between Maoming Han and other 11 Han Chinese populations from Northern and Southern China based on *R*_*st*_ values. **(A)** Multidimensional scaling (MDS) plot between Maoming Han and other 11 Han Chinese populations; **(B)** neighbor-joining (N-J) phylogenetic tree among 12 Han Chinese populations from Southern and Northern mainland China.

From the intercontinental perspective, a MDS was performed between Maoming Han and 21 worldwide populations (Kim et al., 2001; Miranda et al., 2001; Roewer et al., 2005; Mizuno et al., 2008; Alam et al., 2010; Laouina et al., 2011; Wolfgramm Ede et al., 2011; Ramos-Luis et al., 2014; Xu et al., 2015; Han et al., 2016; Rapone et al., 2016; Wang et al., 2017; Alonso Morales et al., 2018; Fan et al., 2018c; Singh et al., 2018; Henry et al., 2019; Lang et al., 2019; Li et al., 2019; Salvador et al., 2019; Reid and Heathfield, 2020). As shown in Supplementary Figure 1, Maoming Han clustered with Han Chinese populations, while other populations got together in accordance with geographical patterns relatively. In addition, to further explore the genetic affinity among Han Chinese populations in mainland China, the degree of differentiation between Maoming Han and Han populations from different administrative divisions of China was assessed by AMOVA and visualized in an MDS plot. Pairwise *R*_*st*_ and corresponding *p* values based on the 17 Yfiler among Maoming Han and 11 other Han Chinese populations from North to South across the mainland China (Yang et al., 2014; Shu et al., 2015; Han et al., 2016; Wang et al., 2016, 2017; Xu et al., 2016; Yao et al., 2016; Jiang et al., 2017; Chen et al., 2018; Fan et al., 2018c; Lang et al., 2019; Yin et al., 2020) are listed in Supplementary Table 5. Significant genetic differences were observed between Maoming Han and all other Han populations (*p* = 0.05) except for Guangdong Han. However, after Bonferroni’s correction (*p* values = 0.05/431 ≈ 0.0001, *n* = 431), there were differences between Maoming Han and Northern Han Chinese from Gansu (*R*_*st*_ = 0.0156, *p* < 0.0001) and Shandong (*R*_*st*_ = 0.0123, *p* < 0.0001), while other Han Chinese populations had no significances with Maoming Han, especially for Guangdong Han (*R*_*st*_ = 0.0008, *p* = 0.1509), indicating the genetic affinity between Southern Han and Northern Han in mainland China. The MDS plot (Figure 2A) based on *R*_*st*_ values clearly demonstrated that the Northern Han populations, Gansu, Jilin, Hebei, Shandong, and Henan (Nanyang), were grouped in the bottom left side, while Hainan, Hubei (Wuhan), Hunan, Guizhou (Zunyi), and Guangdong isolated from the northern cluster and gathered into a Southern Han cluster in the upper right side with Maoming Han.

Subsequently, to make further confirmation about the genetic relationships between Maoming Han and other Han Chinese populations, an N-J phylogenetic tree based on *R*_*st*_ values was constructed (Figure 2B). We found that two main branches could be clearly identified in the N-J phylogenetic tree. The upper branch was Southern Han cluster, which was composed of Maoming, Guangdong, Guangxi, Hainan, Guizhou (Zunyi), Hunan, and Hubei (Wuhan), while Henan (Nanyang), Hebei, Shandong, Gansu, and Jilin got together in the bottom clade as Northern Han cluster. From the perspective of genetics, the analyses above indicated that Maoming Han is a Southern Han population and has relatively close relationships with Guangdong Han, followed by Guangxi Han (*R*_*st*_ = 0.0031, *p* = 0.0155) and Hainan Han (*R*_*st*_ = 0.0033, *p* = 0.0183). The phylogenetic structures of Han Chinese populations from Southern and Northern mainland China in the phylogenetic dendrogram were in line with the results of the MDS. From the geographical scale, Guangdong, Guangxi, Hainan, Guizhou, and Hunan belong to southern administrative divisions of mainland China, while Jilin, Gansu, Shandong, Hebei, and Henan are subordinated to the northern administrative divisions of mainland China (Figure 1). Maoming, lying in the southwest of Guangdong Province, has close geographical distances with Guangxi Zhuang Autonomous Region and Hainan Province. Even though different Han Chinese populations from distinct administrative divisions of mainland China have genetic and linguistic homogeneousness, the genetic distances and population structures of Han Chinese are in accordance with the geographical-scale pattern to a certain extent in mainland China.

### Genetic Affinities and Differentiations Among Maoming Han, Other Han Populations, and Ethnic Minorities From China

According to the history records, the population structures of Maoming Han were mainly affected by the intermarriages with local Tai-kadai language-speaking Baiyue population and the south migrations of Han Chinese from Central China (He, 2012; Chen, 2013)12), while the above population analyses between our studied population and other Southern and Northern Han populations did not hint the relatively intimate relationships between Maoming Han and Han populations of Central Plains. To reveal the genetic structures among Maoming Han, surrounding Han populations and other 16 Chinese ethnic groups (Zhu et al., 2005; Shi et al., 2011; Shan et al., 2014; Zeng et al., 2014; Gao et al., 2015; Guo et al., 2015; Ou et al., 2015; Shu et al., 2015; Bian et al., 2016; Fu et al., 2016; Hu et al., 2017; Wang et al., 2017, 2019; Zhang et al., 2017; Zhao et al., 2017; Chen et al., 2018; Fan et al., 2018a,b,c; Du et al., 2019; Lang et al., 2019; Song et al., 2019; Xie et al., 2019; Ding et al., 2020; Feng et al., 2020; Guan et al., 2020), pairwise *R*_*st*_ and corresponding *p* values were calculated based on 17 Yfiler. As presented in Supplementary Table 6, no difference was observed between Maoming Han and Meizhou Kejia (*R*_*st*_ 0.0007, *p* = 0.2899), while significant genetic differences were observed between Maoming Han and all other Han Chinese or ethnic groups (*p* < 0.05). However, after Bonferroni’s correction (*p* values ≈ 0.0001), there were no differences between Maoming Han and surrounding Han Chinese populations (Supplementary Table 7). Furthermore, we found that Inner Mongolia Daur (*R*_*st*_ = 0.1614), Xinjiang Uighur (*R*_*st*_ = 0.1407), and Lhasa U-Tsang Tibetan (*R*_*st*_ = 0.1294) in Northern China had the longest genetic distances with Maoming Han, while the closest genetic distance was seen in Meizhou Kejia (*R*_*st*_ = 0.0007), followed by Guangxi Han (*R*_*st*_ = 0.0031), Hainan Han (*R*_*st*_ = 0.0033), then by Jieyang Han (*R*_*st*_ = 0.0039), and Chaoshan Han (*R*_*st*_ = 0.0083).

**FIGURE 3:**
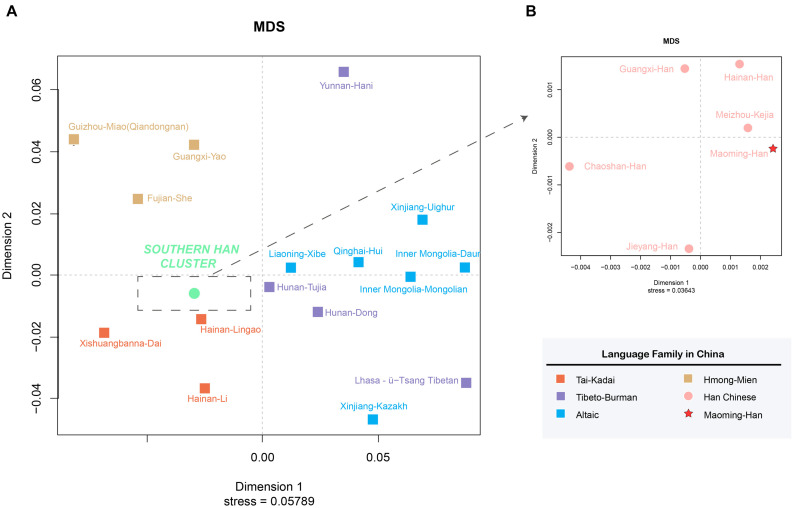
Multidimensional scaling plots among Maoming Han, surrounding Han populations, and Chinese ethnic minorities based on pairwise *R*_*st*_values. **(A)** MDS plot among Maoming Han, five other Chinese Han populations, as well as 16 ethnic minorities from China; **(B)** MDS plot between Maoming Han and surrounding Southern Han Chinese populations.

**FIGURE 4:**
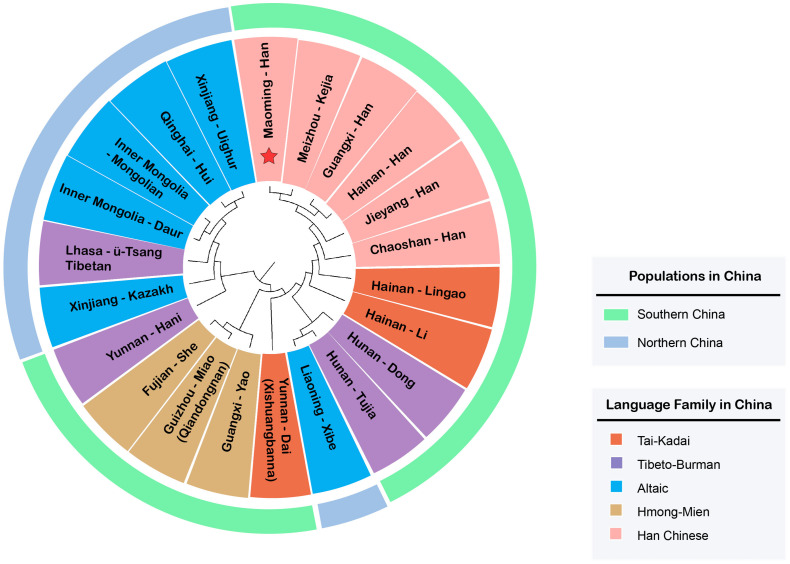
Phylogenetic analysis among the Maoming people, five other Chinese Han populations, as well as 16 reference minorities from China based on pairwise *R*_*st*_ values (the language families and geographic distributions of different populations are displayed in the inner circle and the outer layer, respectively).

On the basis of *R*_*st*_ values of 22 Chinese populations, an MDS plot (Figure 3) and an N-J phylogenetic tree (Figure 4) were performed to depict the forensic genetic landscape of Chinese Han and ethnic groups. As shown in Figure 3A, the Han Chinese populations were closely related to each other and therefore formed a Southern Han cluster, while other 16 minorities were relatively isolated from the Southern Han cluster and dispersed into four main clusters, which were in accord with the distributions of language families in some degree. The Hmong-Mien language-speaking groups, Miao, Yao, and She, clustered together at the upper left, and Dai, Lingao, and Li gathered in the bottom left as the Tai-Kadai language-speaking cluster, while the Tibeto-Burman-language speaking and Altaic-language speaking groups located together at the bottom right with relative separated positions. In addition, Figure 3B indicates the genetic relationships between Maoming Han and surrounding Han populations, which indicated that Maoming Han had a close relationship with Meizhou Kejia. Kejia, also known as Hakka, is a branch of Han Chinese that has a wide distribution in Guangdong Province. The genetic pool of Maoming Han was influenced by the surrounding Han populations, while no direct genetic evidence verified that the Tai-Kadai language-speaking populations contributed to the Maoming Han genetic pool. Furthermore, Meizhou Kejia was first clustered with the Maoming Han, followed by Guangxi Han and Hainan Han, then by Jieyang and Chaoshan in the phylogenetic tree (Figure 4). The tree also revealed that different populations were gathered into two cluster according to their geographical distributions and separated into two main branches: one represented the Altaic language-speaking populations; the other one stood for the Sino-Tibetan language-speaking populations (Han Chinese, Tibeto-Burman, Hmong-Mien, and Tai-Kadai), which was roughly congruent with the results of corresponding MDS (Figure 3).

From the perspective of linguistics, geographies, and genetics, the phylogenetic analyses (both the MDS plots and N-J phylogenetic tree) demonstrated that Maoming Han was isolated from Chinese ethnic minority groups relatively and had a relatively close genetic relationships with Southern Han populations, especially for those with the same dialects and intimate geographical distances (Meizhou Kejia, Guangxi Han, and Hainan Han), which indicated that there might be gene flows between Maoming Han and the surrounding Han populations. In addition, the genetic structures of Han populations correspond to the patterns of the geographical-scale spatial distributions and the relationships of language families. In total, the results of above population genetic analyses indicated that Maoming Han, which is a Southern Han Chinese population, has a relatively close relationship with Meizhou Kejia; therefore, the role of the gene flows from surrounding Han populations in shaping the genetic pool of Maoming Han cannot be ignored.

## Conclusion

In the present study, a total of 431 unrelated Guangdong Maoming Han were investigated using the AmpFLSTR^®^ Yfiler^®^ Plus PCR Amplification Kit (Thermo Fisher Scientific, Waltham, MA, United States). The high-quality 27 Y-STR haplotype data of Maoming Han were obtained and submitted to YHRD with the accession number YA004720. Overall, 263 different alleles were identified across all 27 Y-STRs with the number of distinct alleles from 4 to 55. Allele frequencies varied from 0.0004 to 0.7401, and the lowest and highest estimates of GD corresponded to loci DYS391 (0.4027) and DYS385a/b (0.9596), respectively. Genotyping with the 27 Y-STRs determined 417 distinct haplotypes in the population of Maoming Han, of which 405 (97.12%) were unique. In the first batch of 27 Yfiler data for Maoming Han, nine intermediate alleles were detected at six Y-STR loci; in addition, duplicated or triplicated alleles and null alleles were not observed. Based on the comparisons of forensic parameters for different sets of Y-STRs (17 Yfiler set and 27 Yfiler Plus set), it demonstrated that the improvements of HD and DC are accompanied by the increasing numbers of Y-STRs. The overall HD and DC of 27 Yfiler in Maoming Han were calculated to be 0.9997 and 0.9675, respectively.

From the perspectives of genetics, linguistics, and geographies, different Han Chinese populations from distinct administrative divisions of mainland China have genetic and linguistic homogeneousness, and the genetic distances and population structures of Han Chinese are in accordance with the geographical-scale pattern to a certain extent in mainland China. Maoming Han, a Southern Han population, has a relatively close genetic relationship with Meizhou Kejia, which has the same language family and has intimate geographical distances with Maoming Han, while no exact genetic evidence supports that there are intimate relationships between Maoming Han and Tai-Kadai language-speaking populations and Han populations of Central Plains. At the same time, we found that the genetic structures of Han populations correspond to the patterns of the geographical-scale spatial distributions and the relationships of language families. As a whole, the sufficient systematic efficiencies of AmpFLSTR^®^ Yfiler^®^ Plus PCR Amplification Kit in Maoming Han demonstrated that it can be widely applied in the population of Guangdong Maoming Han for forensic purposes, and Maoming Han, which is a Southern Han Chinese population, and has a relatively close relationship with Meizhou Kejia; therefore, the role of the gene flows from surrounding Han populations in shaping the genetic pool of Maoming Han cannot be ignored.

## Data Availability Statement

The datasets presented in this study can be found in online repositories. The names of the repository/repositories and accession number(s) can be found in the article/Supplementary Material.

## Ethics Statement

The studies involving human participants were reviewed and approved by Medical Ethics Committee of the Hainan Medical University. The patients/participants provided their written informed consent to participate in this study.

## Author Contributions

HF made significant contributions in the conceptualization, resources, software, formal analysis, and project administration. YL and QX made significant contributions in the investigation. LW performed the validation. QX performed the data curation. HF and QX performed the visualization, wrote and prepared the original draft, and reviewed and edited the manuscript. PQ and S-QW made significant contributions in the supervision of the study. PQ acquired funding for the study. All authors reviewed the manuscript.

## Conflict of Interest

The authors declare that the research was conducted in the absence of any commercial or financial relationships that could be construed as a potential conflict of interest. The handling editor declared a past co-authorship with the authors LW and S-QW.

## Footnotes

1 https://yhrd.org/
