# Supplementary material for: Insights Into Forensic Features and Genetic Structures of Guangdong Maoming Han Based on 27 Y-STRs
Source: Front Genet. 2021 Jun 18;12:690504. doi: 10.3389/fgene.2021.690504 (PMC8253533; doi:10.3389/fgene.2021.690504)
Supplement: Supplementary file 1 [file Data_Sheet_1.zip › Image 1.DOCX]

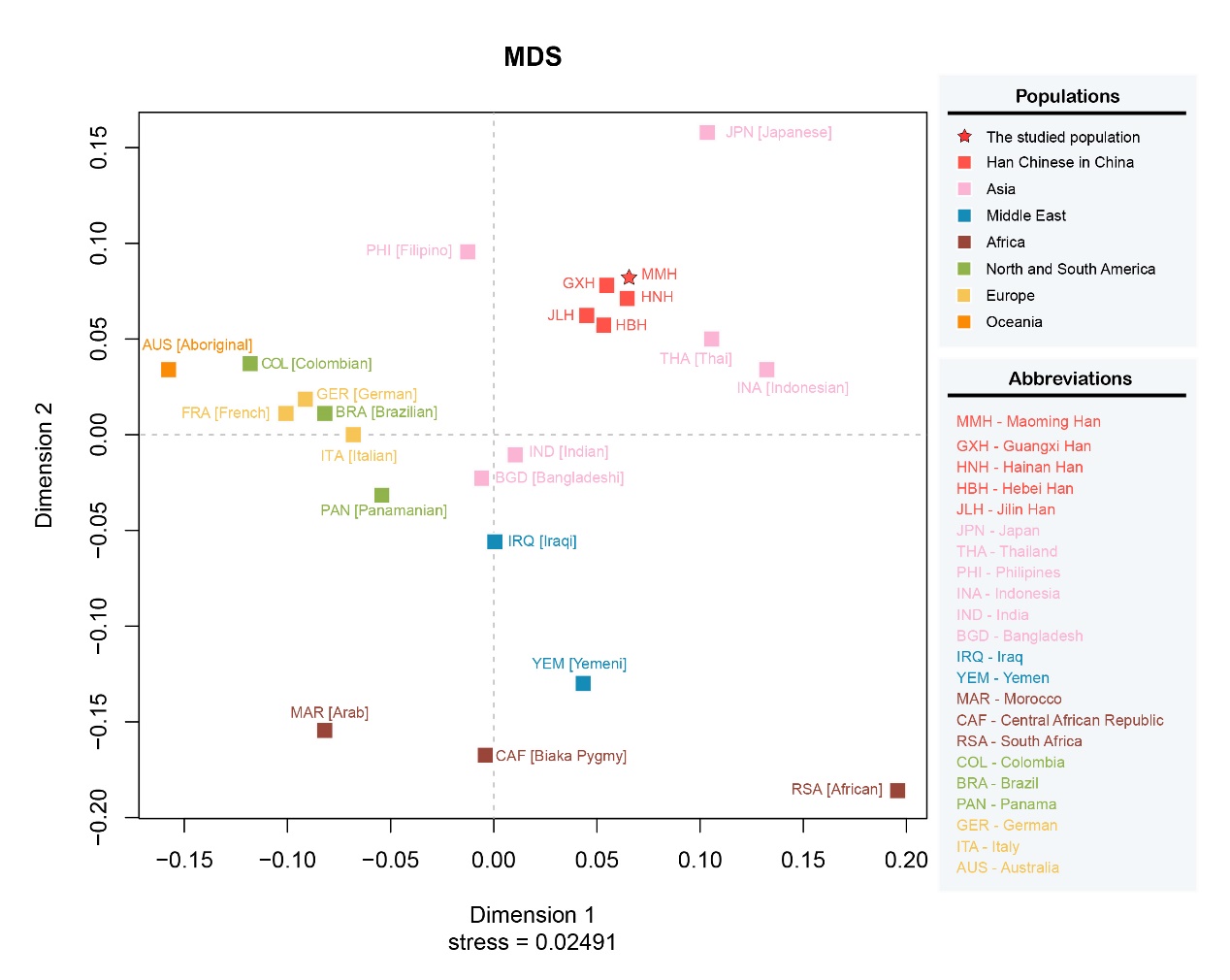


**Supplementary Figure 1 MDS plot between Maoming Han and 21 worldwide populations based on pairwise** $\boldsymbol{R}_{\boldsymbol{st}}$**values.**
